# Perceptions of activity-based offices are associated with employee well-being and self-reported work ability in hybrid work: a cross-sectional study

**DOI:** 10.1093/joccuh/uiaf027

**Published:** 2025-05-20

**Authors:** Elina Tulenheimo-Eklund, Annu Haapakangas, Maria Hirvonen, Virpi Ruohomäki, Kari Reijula

**Affiliations:** Finnish Institute of Occupational Health, P.O. Box 40, FI-00032 Työterveyslaitos, Helsinki, Finland; Department of Public Health, Faculty of Medicine, University of Helsinki, P.O. Box 20, 00014 University of Helsinki, Helsinki, Finland; Finnish Institute of Occupational Health, P.O. Box 40, FI-00032 Työterveyslaitos, Helsinki, Finland; Finnish Institute of Occupational Health, P.O. Box 40, FI-00032 Työterveyslaitos, Helsinki, Finland; Finnish Institute of Occupational Health, P.O. Box 40, FI-00032 Työterveyslaitos, Helsinki, Finland; Finnish Institute of Occupational Health, P.O. Box 40, FI-00032 Työterveyslaitos, Helsinki, Finland; Department of Public Health, Faculty of Medicine, University of Helsinki, P.O. Box 20, 00014 University of Helsinki, Helsinki, Finland

**Keywords:** burnout, hybrid work, occupational health, work engagement, work environment, workplace

## Abstract

**Objectives:**

As digitalization has made knowledge work multilocational, the need for space-efficient, modern-design offices, including activity-based offices, has increased. However, studies of how activity-based offices are related to employee well-being, particularly in hybrid work, are rare. This study investigated whether the perceived activity-based office environment in hybrid work is associated with employee well-being and self-reported work ability.

**Methods:**

We used a cross-sectional survey of 971 employees working in activity-based offices in 5 Finnish organizations in 2022. The workplaces were observed, and the employees responded to a survey of their office environment perceptions, well-being, and work ability. The survey covered burnout risk scores, work engagement, daily recovery, self-reported work ability, insomnia, and pain symptoms. We used the regression analysis general linear model to analyze associations between the environmental perceptions and employee outcomes, adjusting for age, gender, supervisory position, and telework frequency or effort–reward imbalance.

**Results:**

Employees’ daily recovery and self-reported work ability were better when the employees’ office environment perceptions (task privacy, satisfaction with work environment, person-environment fit, workspace support for interaction, ease of workspace switching) were more positive. These associations remained in the adjusted models. Additionally, work engagement increased, and burnout risk scores and insomnia symptoms decreased when workplace conditions were perceived more favorably.

**Conclusions:**

More favorable perceptions of activity-based offices are associated with better employee well-being and self-reported work ability in hybrid work. The perceived office environment seems essential for employee well-being and work ability even when psychosocial aspects (effort–reward imbalance) are taken into account.

Key points:

- **What is already known on this topic:** Flexible, space-efficient, activity-based offices (ABOs) have become more common in knowledge work. ABOs typically have nonassigned workstations in shared open and enclosed workspaces so that employees can change workstations for different work tasks. Office layout can affect employees’ well-being: For example, open-plan offices are prone to more distractions and poorer well-being. However, research on how modern office design is associated with employee health and work ability is lacking.
- **What this study adds:** Favorable perceptions of the ABO environment are related to better employee well-being and work ability in hybrid work. Satisfaction with the office environment and positively perceived task privacy are associated with higher work engagement, better recovery and work ability, lower burnout risk, and fewer insomnia symptoms. How the office environment is perceived is important, as these associations remained even when telework frequency and effort–reward imbalance as a psychosocial factor were accounted for in the analyses.
- **How this study might affect research, practice, or policy:** Employees’ needs and perceptions of premises must be considered when developing the office environment. The perceived environment in ABOs is associated with occupational well-being and work ability, which highlights that occupational health and safety actors’ support is important for organizations and work communities when workspace changes are being made. Employees with reduced work ability may need additional support in ABOs. Our findings emphasize the need for research from the occupational health perspective to explore how employee health and work ability are affected by modern office designs.

## 1. Introduction

New ways of working (NWW) have increased globally. Digitalization and flexible working arrangements have led to multilocational knowledge work, a development accelerated by the COVID-19 pandemic. NWW have led to the introduction of flexible offices, with positive expectations of higher flexibility, support for interaction and productivity, fulfillment of sustainability goals, and a reduction of facility costs.^1^ Less space is needed for hybrid work, which is only partly done in the employer’s premises. Hence, space-efficient and modern activity-based offices (ABOs)^2^ have become more common in knowledge work, which highlights the need for occupational health research focusing on employees working in ABOs.

Flexible ABOs are “open-office environments comprising a variety of additional open, half-open and enclosed activity-related working locations without assigned workstations.”^3^ In ABOs, unlike traditional open-plan offices, employees are expected to switch and choose a workstation that facilitates their current activity and matches their preferences.^1^^,^^3^ The typical features of ABOs, for instance, open main work environment and enclosed working locations, determine architectural privacy, which affects the psychological experience of privacy and perceived office environment, and factors such as control over noise, distractions, and visual privacy.^3^ Office design seems to be a relevant issue for work ability and employee health, as previous research on traditional open-plan offices has found it to be associated with sickness absences,^4^ disability retirement,^5^ difficulties in concentrating,^6^ more symptoms from environmental dissatisfaction, and having more people in a single workspace.^6^^,^^7^ However, the results concerning traditional open-plan offices are not directly applicable to ABOs due to their features that differ from open-plan offices. The employees’ own activity and flexible use of activity-related spaces are basic assumptions of ABOs and may affect how premises suit the users’ needs. According to the person-environment (PE) fit theory, stress evolves from a misfit between person and environment, which in turn leads to strain and efforts to resolve the misfit.^8^ Thus, in ABOs, switching to a more suitable (eg, quiet) space may influence perceived fit and well-being.^3^^,^^8^^,^^9^

ABOs have been considered a promising concept for addressing the work-related needs of both communication and concentration while achieving high space-efficiency. However, despite positive expectations, ABOs’ associations with interaction, social support, and employee performance and well-being have been both positive and negative.^10^^-^^12^ Positive findings may relate to more communication in ABOs, whereas negative findings may be due to difficulties in communicating confidentially or locating colleagues, as well as perceived distractions, poor privacy, and concentration problems that are often reported in ABOs.^10^^,^^11^ Knowledge about the relationship between ABOs and health-related outcomes, for example, mental health or somatic symptoms, and work ability remains very limited.^10^^,^^11^^,^^13^ To our knowledge, few studies have focused on employees’ work ability in ABOs.^2^^,^^14^ The preliminary findings suggest that PE-fit, active workspace switching,^2^ and environmental satisfaction in ABOs^14^ are associated with better work ability. As work ability is dynamic, the work environment can either facilitate or hinder employee performance.^15^ Data on burnout and work engagement in modern offices are also scarce,^12^^,^^16^^,^^17^ even though physical working conditions^18^^,^^19^ and features of office design are assumed to act as job demands or resources.^3^^,^^20^ Generally, high job demands and lack of job resources predict burnout, whereas job resources increase work engagement,^19^ defined as “a positive, fulfilling, work-related state of mind characterized by vigor, dedication, and absorption”.^21^ Some studies have found that distractions in ABOs are associated with increased fatigue or strain^16^^,^^17^ and reduced work engagement.^17^ Work stressors also predict insufficient psychological detachment from work, whereas work resources are related positively to recovery experiences.^22^ Recovery’s association with workspace satisfaction and flexible work in ABOs is tentatively supported by earlier studies.^14^^,^^23^

Despite the increase in hybrid work, most office design research has not considered telework.^2^^,^^4^^,^^16^ Telework may potentially shape user experiences of ABOs^2^ and compensate for adverse conditions at the office,^16^ though the relation seems complex as there are also results from open-plan offices showing no protective effect of telework.^4^ Hence, it is important to take telework into account in office design research in the changing world of work. Moreover, the psychosocial and physical work environment are intertwined: For example, having more occupants in the one office space is associated with more psychosocial work stressors,^7^ and satisfaction with the office environment is linked to workplace social capital.^14^ Psychosocial factors may confound the relations between office design and well-being, and yet few studies have considered this. Some previous findings suggest that office design and environmental satisfaction are associated with employee well-being and work ability even when the psychosocial environment is considered.^6^^,^^7^^,^^14^

Concerning the inconclusive evidence of how modern office design relates to employees’ health and work ability, and to succeed in the use of ABO concept, it is important to clarify how the perceived ABO environment is related to employee well-being. Based on the literature, we focused on privacy and concentration issues, PE-fit, satisfaction with the work environment, workspace switching, and support for interaction as office environment perceptions. Our aim was to examine employees’ perceptions of ABOs in hybrid work and whether these perceptions are associated with well-being at work (work engagement, burnout, recovery), self-reported work ability, insomnia symptoms, and pain. In our analyses we used the person-related variables of gender, age, and supervisory position as potential confounders, because employees’ characteristics may play a role in user behavior and perceptions of ABOs.^2^^,^^3^ We also considered the psychosocial environment and teleworking frequency in the models, due to their potential impact on the perceived office environment^2^^,^^14^ and employee well-being.^4^^,^^7^^,^^16^ As a psychosocial factor we chose the effort–reward imbalance ratio (ERI), that is, the balance between efforts invested and rewards received from work. An imbalance can be harmful to health.^24^^,^^25^ Hence, we also aimed to assess whether the associations between employee perceptions of the ABOs and employee outcomes persisted when we considered teleworking and ERI as a psychosocial factor. Based on the literature reviewed above, we assumed that more favorable perceptions of ABOs are associated with better employee well-being and the associations are independent of the ERI. Given the scarcity and inconsistency of findings concerning telework in this context, the present analyses are explorative in nature. If the associations between the perceptions of the ABOs and employee outcomes persist after accounting for telework, this would indicate that the perceived office environment is also relevant to employee well-being in hybrid work.

**Figure 1:**
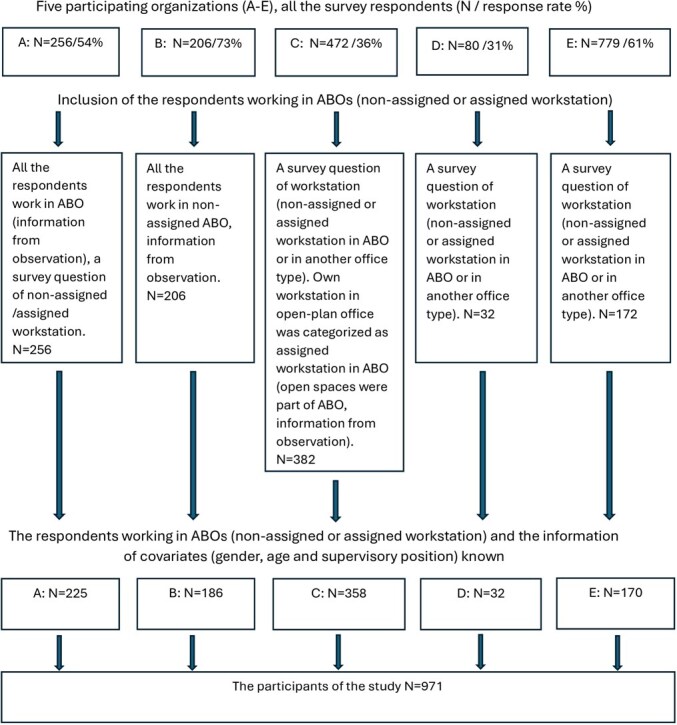
Inclusion criteria for the study. Respondents to the survey were included if they worked in an activity-based office (ABO) and information about gender, age, and any supervisory position was provided.

## 2. Methods

### 2.1. Study design and data collection

From August to November 2022, we collected cross-sectional survey data on employees working in the ABOs of 5 organizations in southern Finland. We also used information from workspace observations. The survey was part of a larger project on different office types and their associations with employee well-being and sick leave.

The organizations were recruited between November 2020 and February 2021. Either the entire organization or certain units of it participated in the study. We emailed the online survey link to all the individuals working in the participating units.

The participants voluntarily gave their written informed consent to participation and responded to the survey. The Ethical Review Board of the Finnish Institute of Occupational Health in Helsinki, Finland approved this study in advance.

**Table 1:** Descriptive statistics of participants.

**Variable**	***n***	**Mean (SD)**	**Frequency, %**
**Age**	971	48.0 (10.1)	
**Gender**	971		
**Male**	246		25.3
**Female**	725		74.7
**Other**	0		0.0
**Supervisory position**	971		
**Yes**	117		12.0
**No**	854		88.0
**Education**	799		
**General upper secondary school, high school or lower education**	43		5.4
**Post-secondary education**	104		13.0
**Bachelor’s degree (of applied sciences or similar)**	131		16.4
**Master’s degree (or similar)**	395		49.4
**Postgraduate (Licentiate or Doctor)**	126		15.8
**Workstation in ABO**	971		
**Shared**	866		89.2
**Assigned**	105		10.8
**Telework frequency**	971		
**Daily**	231		23.8
**3-4 d/wk**	513		52.8
**1-2 d/wk**	121		12.5
**2-3 d/mo; less frequently; I don’t do any remote work**	106		10.9
**Effort–reward imbalance, ratio 0.2-5.0**	963	1.3 (0.5)	
**Perceived activity-based office environment**			
**Task privacy, scale 1-7**	966	4.2 (1.5)	
**Satisfaction with work environment, scale 1-7**	963	4.7 (1.6)	
**Person-environment fit**	798		
**Strongly agree/more or less agree**	527		66.0
**Neither agree nor disagree**	103		12.9
**More or less disagree/strongly disagree**	168		21.1
**Workspace support for interaction**	961		
**Strongly agree/more or less agree**	553		57.5
**Neither agree nor disagree**	224		23.3
**More or less disagree/strongly disagree**	184		19.1
**Ease of workspace switching**	790		
**Very easy/quite easy**	416		52.7
**Neither easy nor difficult**	162		20.5
**Quite difficult/very difficult**	212		26.8
**Access to quiet workspaces**	800		
**Yes, sufficiently**	318		39.8
**Varying, not well enough**	448		56.0
**No, not at all**	34		4.3
**Outcome variables**			
**Work engagement, scale 0-6**	965	4.5 (1.2)	
**Burnout risk scores, scale 1-5**	799	2.1 (0.5)	
**Daily recovery, scale 0-10**	966	7.1 (2.1)	
**Self-reported work ability (Work Ability Score), scale 0-10**	967	8.1 (1.5)	
**Insomnia symptoms, scale 1-5**	800	2.3 (0.9)	
**Pain index, scale 0-100**	797	75.0 (21.1)	

### 2.2. Participating workplaces

The organizations were statutory operators, 1 of which operated in the Finnish municipal sector. Their personnel were mostly female. At the time of the survey, at least part of the organizations’ personnel had used ABOs from 1 to several years. The research team observed the workplaces on site and gathered information on how they were used. The ABOs included: open-plan areas; quiet areas or nonassigned rooms for individual work; meeting rooms; back-up spaces for withdrawal, conversations, or virtual meetings; break rooms; and storage facilities for documents and personal items. The offices were mainly of good quality and renovated or built in the 21st century. Workstations included mainly adjustable sit-stand desks and a computer screen to connect to a laptop, and many workstations had screens surrounding the desks to improve acoustics and privacy. Some ABOs had ergonomic tools and different work chair options. We observed that the acoustics (eg, the intelligibility of speech, the acoustic materials) varied in quality. Workstations were mainly nonassigned. The occupancy rate of the premises was still low after the extensive teleworking during the COVID-19 pandemic, and in some units the employees had occupied general back-up spaces for their own use. The teleworking rules were flexible in all the organizations, with some variation depending on tasks and recommendations to be present at the office. Tasks that required daily presence at the workplace were not usually done in ABOs.

### 2.3. Participants

The participants worked in many different knowledge work occupations, ranging from researchers to administrative personnel, IT specialists, and economists. All (*n* = 971) worked in ABOs, mainly at nonassigned workstations (89.2%). Figure 1 presents the study inclusion criteria; survey information and workspace observations were used. The response rate in the organizations was between 31% and 73%. The participants were highly educated (see Table 1), their mean age was 48.0 years, and the majority were female (74.7%). Twelve percent worked in supervisory positions.

### 2.4. Measures

Our project survey covered several items, of which we used some in this study (see Table 2; the Supplementary material contains complete descriptions of the questions used). One organization’s survey (Figure 1, organization E) was combined with the Finnish Public Sector Study and did not include all the items.

To elicit the participants’ perceptions of the office environment, we measured task privacy in terms of distractions and ability to concentrate at the workplace (adapted from Oldham^26^), satisfaction with work environment,^27^ PE-fit,^28^ workspace support for interaction,^28^ and ease of workspace switching. The respondents also rated their perceived availability of quiet workspaces for work requiring concentration (adapted from Bodin Danielsson and Theorell^29^).

### 2.5. Outcomes

We measured work engagement with a 3-item version of the Utrecht Work Engagement Scale (UWES-3),^30^ and burnout risk using a clinically validated Burnout Assessment Tool—the BAT-12.^31^^,^^32^ This contains questions on the 4 dimensions of burnout syndrome—exhaustion, mental distancing, cognitive impairment, and emotional impairment.^31^^,^^32^ Higher mean scores of the 12 items on a continuous scale indicate more symptoms and a higher risk of burnout. The survey also included a single item of daily recovery from the strain of the working day.^33^

Self-reported work ability was elicited by a single question on current work ability (Work Ability Score, WAS),^34^ which is part of the larger Work Ability Index (WAI).^35^ This single item associates strongly with WAI and predicts health-related outcomes and sick leave.^34^ Insomnia symptoms over the last 4 weeks were elicited by 4 questions modified and adapted from an item of self-reported insomnia^36^; the description of nonorganic insomnia symptoms in the International Statistical Classification of Diseases and Related Health Problems (ICD-10) was also considered. The respondents assessed how often they had trouble falling asleep, woke up at night and had difficulties or failed to fall asleep again, had unrefreshing sleep, and how often their daytime condition or functional capacity was affected by their sleep problems. We calculated the mean of the 4 items to indicate self-reported insomnia symptoms. The respondents evaluated their pain over the last 4 weeks using a 2-item measure (1 dimension of the RAND-36-Item Health Survey).^37^ We recoded the responses to the index of the bodily pain subscale 0-100 and calculated the mean of the 2 items according to the RAND-36 measure so that the higher scores indicated less experienced pain.

**Table 2:** Variables for perceptions of the activity-based offices and study outcomes.

**Variable**	**Measure**	**Scale**
**Perceived activity-based office environment**		
Task privacy, adapted from Oldham	3-item measure; eg, When I am at the office, I can work with few distractions or interruptions	7-point scale: 1 strongly disagree to 7 strongly agree. Mean score 1-7
Satisfaction with work environment	How satisfied are you with your work environment as a whole (at the workplace)?	7-point scale: 1 very dissatisfied to 7 very satisfied
Person-environment fit Workspace support for interaction	The work premises are well-suited for carrying out my work tasks. The work premises support interaction between individuals	Scale 1-5: 1 strongly disagree/more or less disagree/neither agree nor disagree/more or less agree/5 strongly agree
Ease of workspace switching	How easy is it for you to find a more suitable workspace during a working day and go there, eg, if you need to concentrate or have a confidential discussion or phone call?	Scale 1-5: 1 very difficult/quite difficult/neither easy nor difficult/quite easy/5 very easy
Availability of quiet workspaces, adapted from Bodin Danielsson and Theorell	At the office, do you have access to a quiet workspace for concentrated work?	Scale 1-3: 1 no, not at all/varyingly, not well enough/3 yes sufficiently
**Outcomes**		
Work engagement, Ultra-Short Measure for Work Engagement, UWES-3	3-item measure, eg, I feel full of energy when I am working	Scale 0-6: 0 never/a few times a year/once a month/a few times a month/once a week/a few times a week/6 daily. Mean score 0-6
Burnout risk scores, Burnout Assessment Tool, BAT-12	12-item measure, eg At work, I feel mentally exhausted	5-point scale: 1 never/rarely/sometimes/often/5 always. Mean score 1-5
Daily recovery	Do you recover from the strain of the working day before the next day?	Scale 0-10: 0 not at all to 10 completely
Self-reported work ability, a single item (WAS)	Let’s assume that your work ability at its all-time best would be given 10 points, and 0 points would indicate that you are completely unable to work. What point score would you give your current work ability?	Scale 0-10
Insomnia symptoms	Four questions on insomnia symptoms over last 4 weeks, eg, Have you had trouble falling asleep?	Scale 1-5: 1 less frequently than once in 4 weeks or never/less frequently than once a week/1-2 days a week/3-5 days a week/5 daily or almost daily. Mean score 1-5
Pain index, subscale of RAND-36-Item Health Survey	Two questions to evaluate bodily pain over last 4 weeks (see details in Supplementary data)	Scale 0-100 (scale of the sum variable; see response options in Supplementary data)

Abbreviation: WAS, Work Ability Score.

### 2.6. Covariates

Age, gender, and supervisory position were included as covariates. We elicited telework frequency by asking how often the respondents normally worked remotely, combining 6 response options into 4 categories (daily; 3-4 d/wk; 1-2 d/wk; 2-3 d/mo, or less frequently or no remote work; adapted from Ruohomäki et al.^38^). As a psychosocial factor we used a 4-item ERI measure^24^ that contained 3 questions on rewards from work in terms of income and employee benefits, received recognition, and personal satisfaction; and 1 question on effort, that is, investment into work (5-point scale from 1 very little, to 5 a very great extent). The ERI ratio of the effort score and the individual mean of the 3 reward items was then calculated, and an ERI ratio above 1.0 showed greater experienced imbalance.

### 2.7. Data analysis

We coded all the variables concerning the perceptions of ABOs so that the positive perceptions had higher scores. In the analyses of the PE-fit, workspace support for interaction, and ease of workspace switching, the 5-point scales were recoded into 3 categories to facilitate the presentation and interpretation of the results and also because the outermost negative responses were rare. In these variables, “agree” was reported as a good perceived PE-fit and workspace support for interaction. All the sum variables required a response to more than half of the items and we calculated the mean score of the items using the available individual responses. The item nonresponse rate was low. The number of observations in the variables varied between 790 and 971, due to some missing individual responses and, for some questions, lack of data from the municipal organization.

As a preliminary data analysis, we conducted pairwise correlation analysis, and calculated Cronbach alpha coefficients for the sum variables (Table 3). We analyzed the data using the general linear model (GLM) regression analysis in SAS for Windows 9.4. The figures presenting the regression model results were constructed using RStudio 2023.03.1 for Windows.

**Table 3:** Correlations between variables analyzed in this study and Cronbach alpha values for sum variables.^a^

	**Cronbach alpha**	**1**	**2**	**3**	**4**	**5**	**6**	**7**	**8**	**9**	**10**	**11**	**12**	**13**	**14**	**15**	**16**	**17**
**1 Task privacy**	.81	1.00																
**2 Satisfaction with work environment**	—	0.70^***^	1.00															
**3 Person-environment fit**	—	0.55^***^	0.66^***^	1.00														
**4 Workspace support for interaction**	—	0.31^***^	0.46^***^	0.47^***^	1.00													
**5 Ease of workspace switching**	—	0.52^***^	0.60^***^	0.51^***^	0.37^***^	1.00												
**6 Access to quiet workspaces**	—	0.51^***^	0.53^***^	0.43^***^	0.31^***^	0.57^***^	1.00											
**7 Work engagement**	.87	0.14^***^	0.19^***^	0.07	0.09^**^	0.04	−0.01	1.00										
**8 Burnout risk scores**	.87	−0.23^***^	−0.24^***^	−0.14^***^	−0.14^***^	−0.14^***^	−0.11^**^	−0.51^***^	1.00									
**9 Daily recovery**	—	0.24^***^	0.23^***^	0.18^***^	0.15^***^	0.18^***^	0.14^***^	0.38^***^	−0.62^***^	1.00								
**10 Self-reported work ability**	—	0.23^***^	0.23^***^	0.14^***^	0.16^***^	0.15^***^	0.10^**^	0.47^***^	−0.66^***^	0.64^***^	1.00							
**11 Insomnia symptoms**	.81	−0.18^***^	−0.17^***^	−0.11^**^	−0.07^*^	−0.05	−0.09^*^	−0.26^***^	0.48^***^	−0.46^***^	−0.46^***^	1.00						
**12 Pain index**	.83	0.07^*^	0.08^*^	0.04	0.06	0.09^**^	0.06	0.11^**^	−0.29^***^	0.21^***^	0.28^***^	−0.28^***^	1.00					
**13 Age**	—	−0.09^**^	−0.14^***^	−0.12^***^	−0.09^**^	−0.04	−0.01	0.07^*^	−0.00	0.05	0.00	−0.08^*^	−0.01	1.00				
**14 Gender, male**	—	0.16^***^	0.05	0.02	0.02	0.10^**^	0.11^**^	−0.09^**^	−0.05	0.03	−0.01	0.01	0.06	0.02	1.00			
**15 Supervisory position, yes**	—	0.10^**^	0.10^**^	0.09^**^	0.06	0.10^**^	0.13^***^	0.06	−0.05	0.02	0.06	−0.02	0.02	0.11^***^	0.12^***^	1.00		
**16 Telework frequency**	—	−0.29^***^	−0.22^***^	−0.15^***^	−0.15^***^	−0.16^***^	−0.09^**^	−0.02	0.06	0.04	−0.04	0.03	−0.04	0.04	−0.11^***^	−0.16^***^	1.00	
**17 Effort–reward imbalance** ^b^	.70	−0.27^***^	−0.22^***^	−0.16^***^	−0.12^***^	−0.19^***^	−0.20^***^	−0.19^***^	0.32^***^	−0.43^***^	−0.25^***^	0.25^***^	−0.09^**^	−0.01	−0.14^***^	−0.09^**^	0.02	1.00

a Pearson correlation coefficients for associations between continuous variables, and Spearman correlation coefficients for associations including categorical variables. Number of observations in pairwise correlation analyses varied between 784 and 971. ^*^P < .05; ^**^P < .01; ^***^P < .001.

b The Cronbach alpha value was calculated only for the reward items.

**Figure 2:**
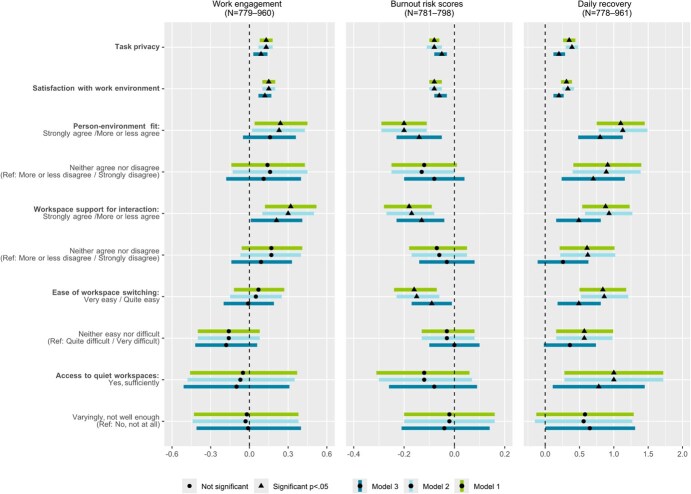
Well-being at work. Results of the regression model concerning work engagement, burnout risk scores, and daily recovery with unstandardized adjusted estimates, 95% CIs, and *P* values. Each outcome was analyzed with each predictor variable in separate analyses with Model 1-3 covariates. Model 1 analyses were adjusted for age, gender, and supervisory position; telework frequency was added to Model 2 analyses. Model 3 analyses were adjusted for Model 1 covariates and the effort–reward imbalance index.

**Figure 3:**
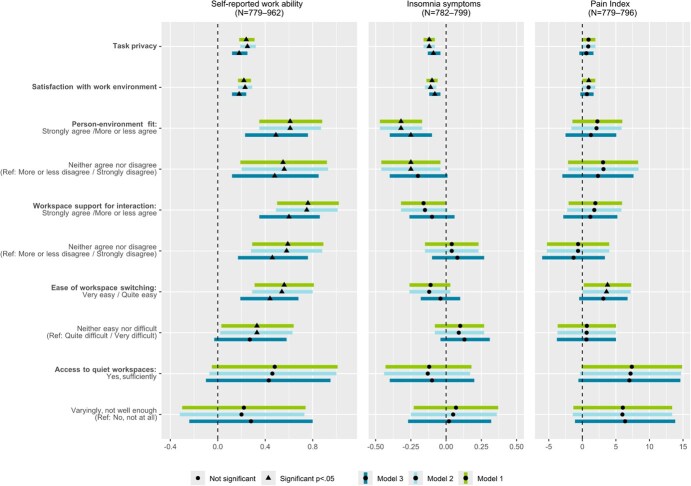
Self-reported work ability and symptoms. Results of the regression model concerning self-reported work ability, insomnia symptoms, and pain index with unstandardized adjusted estimates, 95% CIs, and *P* values. Each outcome was analyzed with each predictor variable in separate analyses with Model 1-3 covariates. Model 1 analyses were adjusted for age, gender, and supervisory position; telework frequency was added to Model 2 analyses. Model 3 analyses were adjusted for Model 1 covariates and the effort–reward imbalance index.

Because the study included different organizations, we evaluated a random effect by estimating the intraclass correlation coefficient (ICC). As this was close to zero, multilevel modeling was not necessary. We analyzed how each office environment perception was associated with each outcome, adjusting for age, gender, and supervisory position (Model 1 covariates). Hence, the results of each outcome in each model included several analyses with different predictors, as the number of observations substantially differed between the predictors. Missing cases were excluded in the GLM analyses, and no imputation was performed. In Model 2, we additionally adjusted the analyses for frequency of teleworking. Finally, in Model 3 we added the ERI ratio in addition to the Model 1 covariates. In Model 3, we excluded teleworking frequency, as this was mainly statistically nonsignificant in Model 2 and had no clear effect on the GLM model results. The simpler models without telework (Model 3) fitted better. Also, in pairwise correlation analyses telework frequency showed no correlation with employee well-being outcomes. We report unstandardized adjusted estimates with 95% CIs (Figures 2 and 3). The alpha level was set at *P* < .05. The unstandardized estimates with 95% CIs and the alpha levels of GLM analyses are shown in detail in the Supplementary data.

## 3. Results

Telework was common: 89.1% of the respondents teleworked at least once a week (Table 1). Most participants (66.0%) experienced a good PE-fit in their ABO. Over half of the respondents agreed that the workspace supported interaction (57.5%), but considered access to quiet workspaces inadequate (60.3%). The mean of self-reported WAS was 8.1 (SD 1.5). The correlations between the variables reflecting environmental perceptions were positive (Table 3). Male gender and supervisory position correlated positively with some perceptions of the ABOs: For example, access to quiet workspaces was perceived more favorably.

### 3.1. Well-being at work

In the regression analyses adjusted for the Model 1 covariates, work engagement had a statistically significant positive association with task privacy, satisfaction with the work environment, good PE-fit, and workspace support for interaction (see Figure 2; more details in Supplementary data). Thus, work engagement increased with employees’ favorable experiences of ABOs. However, ease of workspace switching and access to quiet workspaces were not significantly associated with work engagement in Model 1. Adjustments for telework (Model 2) did not change these results. In the Model 3 analyses (ERI and Model 1 covariates), task privacy, satisfaction with the work environment, and good workspace support for interaction were associated with higher work engagement, but PE-fit, ease of workspace switching, and access to quiet workspaces were not statistically significant predictors of work engagement.

In the Model 1 analyses (Figure 2), the burnout risk scores were lower with higher task privacy and satisfaction with the work environment, good PE-fit and workspace support for interaction, and easy workspace switching. The same environmental perceptions as those in Model 1 were associated with a lower burnout risk in the analyses of Models 2 and 3. Perceived access to quiet workspaces was not significantly associated with burnout risk scores in any of the models. Daily recovery increased along with higher task privacy and satisfaction with the work environment, good PE-fit and workspace support for interaction, easy workspace switching, and sufficient access to quiet workspaces in all the tested Models 1-3 (Figure 2).

### 3.2. Self-reported work ability and symptoms

Self-reported work ability increased along with higher task privacy, satisfaction with the work environment, good PE-fit, workspace support for interaction, and easy workspace switching in Models 1, 2, and 3 (Figure 3).

The higher the reported task privacy, satisfaction with the work environment, and good PE-fit in all the models, the fewer were the insomnia symptoms (Figure 3). Higher ratings of workspace support for interaction showed a nonsignificant tendency toward fewer insomnia symptoms in Models 1 and 2. Ease of workspace switching and access to quiet workspaces were not significant predictors of insomnia symptoms.

The respondents reported less pain when their satisfaction with the work environment was higher and workspace switching was easy (Model 1, Figure 3). In Model 2, less pain was also reported when workspace switching was perceived as easy, whereas other perceptions of the ABOs had no statistically significant associations with pain. None of the environmental perceptions showed statistical significance for pain when ERI was added (Model 3), though there were tendencies toward less pain with easy workspace switching and sufficient access to quiet workspaces (Figure 3).

Telework frequency was mainly nonsignificant in the analyses, but the ERI showed statistical significance (*P* < .05) for all outcomes, being negatively related to employee well-being. The *R*^2^ values of the models with statistically significant findings regarding environmental perceptions as predictors varied from 0.01 to 0.06 in the analyses of Model 1, 0.01-0.08 in Model 2, and 0.06-0.21 in Model 3, the highest being for daily recovery and the lowest for pain.

## 4. Discussion

This study analyzed employees’ perceptions of ABOs, and whether these were associated with well-being at work, self-reported work ability, insomnia symptoms, and pain. When employees perceived the ABOs positively, burnout risk scores were lower, and work engagement and daily recovery increased. Moreover, when perceptions of the ABOs were favorable, work ability was better and fewer insomnia symptoms were reported.

Our study strengthens the understanding that ABOs are related to employee well-being, as we took into account both the psychosocial environment and telework, which previous research has neglected. The workspace observations in our study provided objective information on the ABOs and improved the reliability of workspace selection. The results imply that successful office design may potentially enhance occupational well-being, as work engagement increased along with favorably perceived ABO environment. However, a few studies have observed a decrease in work engagement after moving to an ABO.^12^^,^^17^ The previous findings are probably related to perceived distractions,^17^ and could reflect adaptation or a more permanent change after relocation.^12^ Although these studies are in line with our findings in reporting an association between office design and work engagement, they concern comparisons of different office designs. Our results, on the contrary, show that the perceptions of work environment vary within the ABO design and are associated with work engagement. Our study provides insights into the application of the Job Demands - Resources model^18^ in understanding the physical environment: First, it suggests that work environments also involve positive aspects, for instance good privacy, that is, better control of distractions in premises, and support for interaction may act as job resources enhancing engagement.^3^ Hence, such resource-based mechanisms warrant more attention in future research, in contrast to largely risk-related earlier approaches. Second, the variation in the perceived work environment within the same office design points to the importance of also considering individual and task-related factors, as well as job crafting,^18^ as means to improve an individual’s own job resources, both in future research and in practice. The present findings are important for organizations implementing the ABO concept, as work engagement is positively associated with organizational outcomes such as commitment.^19^

Our findings of better task privacy and lower burnout risk scores capture comprehensively the different dimensions of burnout, but are not surprising in light of previous results that have shown associations between more exhaustion and perceived distractions in ABOs.^16^^,^^17^ As the well-being of the respondents was good on average (Table 1), the perceived changes in the burnout risk score estimates (Figure 2) were below the general cut-off points for probable severe burnout.^32^ The findings emphasize the importance of sufficient task privacy in offices. However, closed spaces to help concentration are often scarce in ABOs in proportion to the amount of high-complexity tasks performed.^9^ Surprisingly, we found no significant association between access to quiet workspaces and burnout risk. Perceived task privacy is likely to be a more sensitive measure and reflects more individual experiences of disturbances at work. Furthermore, employees with burnout symptoms may be more sensitive to distractions, as cognitive impairment is typical in burnout.^32^ The finding that burnout risk was also related to diverse perceptions of ABO was novel.

As another key finding, daily recovery was related to all the analyzed perceptions of ABO, including the availability of quiet workspaces, in all the models. Our observations regarding recovery and satisfaction with the work environment were in line with those in earlier research.^14^ Better self-reported work ability was also related to several positive perceptions of ABOs, which increases our knowledge of the association between work ability and PE-fit, user behavior,^2^ and environmental satisfaction in ABOs.^14^ Additionally, we found that a favorably perceived office environment was linked to fewer insomnia symptoms. Previous research on insomnia symptoms among ABO employees is very limited, though work demands and control are generally associated with sleep disturbances.^39^ A recent study has also shown that when self-rated telework environment was poor, more overall somatic symptoms, including insomnia, were reported.^40^

Most associations between the perceived ABO environment and employee well-being persisted when teleworking was added to the analyses. So far, office design research has largely overlooked telework. This finding emphasizes that the office environment plays a role in employee well-being in hybrid work also. The results also showed consistent associations between the perceived ABO environment and well-being, even when psychosocial aspects were included. Compared with employee perceptions of the ABOs, the ERI was a stronger predictor of many well-being outcomes, which was expected.^7^^,^^24^ However, office design and how the office environment is perceived seem to play an independent role in employee work ability and well-being.^7^^,^^14^ The office environment might even explain the higher risk of sickness absences and disability retirement, as associations with shared traditional office designs have been found.^4^^,^^5^ Office design may also shape the psychosocial work environment.^3^^,^^10^ Our results agree that it is essential to consider psychosocial factors when investigating the relationship between office design and employee well-being.^7^^,^^14^

Our findings emphasize the role of occupational health’s support when organizations redesign the office environment. Several factors of the perceived ABO environment, including support for interaction, good task privacy, and PE-fit, may potentially promote employees’ work ability and recovery. Employees with decreased work ability and poor recovery may also find working in ABOs difficult, especially in open spaces with more distractions. For them, workspace switching may be challenging, which is problematic as switching behavior is a basic assumption in the design of ABOs.^1^ Working on high-complexity tasks in an open space may lead to a misfit, and eventually impair performance and employee well-being.^8^^,^^9^ This is also a risk for other employees as well as those with decreased work ability, because passive switching is common in ABOs.^1^

For the change to an ABO to be successful, employees’ work ability and special needs must be considered, along with analyses of different tasks and job profiles during the design process. Employees’ active participation and a user-centric approach during the planning and implementation phases may contribute to creating functional premises. Moreover, continuous improvement of premises and related practices, while listening to the workspace users’ feedback, is important. Nevertheless, the ABO concept may not be the optimal solution for all office work, such as tasks with low variety or high concentration needs. The options for work requiring great concentration and sufficient task privacy at the workplace require special attention, as previously underlined.^9^^,^^17^ In ABOs, this can be supported by an adequate number of closed spaces for individual work and quiet zones in open areas. Additionally, the policies discussed for office use are important, and encouraging employees to actively use various workspaces and addressing potential hindrances to flexibly switch spaces deserves attention. Preventive occupational health expertise can benefit organizations during workspace changes by providing information on health and work ability perspectives when planning and assessing potential workspace solutions. Employees with decreased work ability may also need individual support in ABOs.

The limitations of our study include its cross-sectional design, which does not allow conclusions about causality. The study used self-reported measures, and additional objective measures of both the work environment and employee health might provide more insight into the relation between office design and well-being. Factors such as employee health or unknown psychosocial factors may also have confounded the results. Missing data may have caused potential bias, although the results showed consistent associations between the analyzed predictors and outcomes, even when the number of observations differed due to missing cases. The findings concern knowledge work in ABOs in Finland, which may limit generalizability to other workplace environments and cultures. As the occupancy rates of the premises were low, the participants’ experiences of ABOs may have been different from those in more typical situations.

### 4.1 Conclusions

This study provided novel evidence that the diverse perceptions of ABO working conditions are associated with self-reported work ability and well-being in hybrid work. Poorly perceived ABO environments showed an adverse relation with work ability, also when the psychosocial work environment was taken into account. Favorably perceived office environments with, for example, good task privacy and support for interaction, may potentially promote work engagement, recovery, and work ability. It is important that employees’ needs are taken into account when workspaces are renewed. Organizations could benefit from support provided by occupational health in ABO implementation and in evaluating the premises’ significance for employee health. Subgroups with decreased work ability should receive special consideration in office design. Longitudinal studies with objective measures and register-based data could provide essential information for weighing the benefits and risks of the ABO concept.

## Supplementary Material

Web_Material_uiaf027

## Data Availability

The data for this article cannot be shared publicly due to protection of the personal data and privacy of the participants.
