# Supplementary material for: Perceptions of activity-based offices are associated with employee well-being and self-reported work ability in hybrid work: a cross-sectional study
Source: J Occup Health. 2025 May 20;67(1):uiaf027. doi: 10.1093/joccuh/uiaf027 (PMC12131161; doi:10.1093/joccuh/uiaf027)
Supplement: Web_Material_uiaf027 [file web_material_uiaf027.zip › Supplementary_Data_File 2.docx]

**Supplementary Data, File 2. Results from regression models**

Tulenheimo-Eklund E., Haapakangas A., Hirvonen M., Ruohomäki V., Reijula K. Perceptions of activity-based offices are associated with employee well-being and self-reported work ability in hybrid work: A cross-sectional study. Journal of Occupational Health.

| **Supplementary Table 1.** Results from regression models for work engagement and burnout risk scores with unstandardized adjusted estimates and 95% confidence limits (CL) and p-values. | | | | | | | | | |
| --- | --- | --- | --- | --- | --- | --- | --- | --- | --- |
| **Outcome: Work engagement** | **Model 1** (N=786–960) | | | **Model 2** (N=786–960) | | | **Model 3** (N= 779–953) | | |
|  | **Estimate** | **95% CL** | **p** | **Estimate** | **95% CL** | **p** | **Estimate** | **95% CL** | **p** |
| **Task privacy**** | 0.13 | 0.08; 0.18 | <.001 | 0.13 | 0.07; 0.18 | <.001 | 0.09 | 0.03; 0.14 | 0.001 |
| **Satisfaction with work environment**** | 0.15 | 0.10; 0.20 | <.001 | 0.15 | 0.10; 0.20 | <.001 | 0.12 | 0.07; 0.17 | <.001 |
| **Person-environment fit*** |  |  |  |  |  |  |  |  |  |
| Strongly agree /More or less agree | 0.24 | 0.04; 0.45 | 0.021 | 0.23 | 0.02; 0.43 | 0.030 | 0.16 | -0.05; 0.36 | 0.131 |
| Neither agree nor disagree | 0.14 | -0.14; 0.43 | 0.328 | 0.16 | -0.13; 0.45 | 0.283 | 0.11 | -0.18; 0.40 | 0.457 |
| More or less disagree / Strongly disagree | ref | - |  | ref | - |  | ref | - |  |
| **Workspace support for interaction**** |  |  |  |  |  |  |  |  |  |
| Strongly agree /More or less agree | 0.32 | 0.12; 0.52 | 0.002 | 0.30 | 0.10; 0.50 | 0.004 | 0.21 | 0.01; 0.41 | 0.040 |
| Neither agree nor disagree | 0.17 | -0.06; 0.41 | 0.144 | 0.17 | -0.07; 0.40 | 0.163 | 0.09 | -0.14; 0.33 | 0.431 |
| More or less disagree / Strongly disagree | ref | - |  | ref | - |  | ref | - |  |
| **Ease of workspace switching*** |  |  |  |  |  |  |  |  |  |
| Very easy / Quite easy | 0.07 | -0.12; 0.27 | 0.451 | 0.05 | -0.15; 0.25 | 0.628 | -0.01 | -0.20; 0.19 | 0.947 |
| Neither easy nor difficult | -0.16 | -0.40; 0.08 | 0.199 | -0.16 | -0.40; 0.08 | 0.189 | -0.18 | -0.42; 0.06 | 0.139 |
| Quite difficult / Very difficult | ref | - |  | ref | - |  | ref | - |  |
| **Access to quiet workspaces*** |  |  |  |  |  |  |  |  |  |
| Yes, sufficiently | -0.05 | -0.46; 0.37 | 0.820 | -0.07 | -0.48; 0.35 | 0.749 | -0.10 | -0.51; 0.31 | 0.640 |
| Varyingly, not well enough | -0.02 | -0.43; 0.38 | 0.909 | -0.03 | -0.44; 0.38 | 0.883 | -0.01 | -0.41; 0.40 | 0.974 |
| No, not at all | ref | - |  | ref | - |  | ref | - |  |
| **Outcome: Burnout risk scores** | **Model 1** (N=788–798) | | | **Model 2** (N=788–798) | | | **Model 3** (N= 781–791) | | |
|  | **Estimate** | **95% CL** | **p** | **Estimate** | **95% CL** | **p** | **Estimate** | **95% CL** | **p** |
| **Task privacy** | -0.08 | -0.10; -0.06 | <.001 | -0.08 | -0.11; -0.05 | <.001 | -0.05 | -0.08; -0.03 | <.001 |
| **Satisfaction with work environment** | -0.08 | -0.10; -0.05 | <.001 | -0.08 | -0.10; -0.05 | <.001 | -0.06 | -0.08; -0.03 | <.001 |
| **Person-environment fit** |  |  |  |  |  |  |  |  |  |
| Strongly agree /More or less agree | -0.20 | -0.29; -0.11 | <.001 | -0.20 | -0.29; -0.11 | <.001 | -0.14 | -0.23; -0.05 | 0.002 |
| Neither agree nor disagree | -0.12 | -0.25; 0.01 | 0.061 | -0.13 | -0.25; 0.00 | 0.052 | -0.08 | -0.20; 0.04 | 0.205 |
| More or less disagree / Strongly disagree | ref | - |  | ref | - |  | ref | - |  |
| **Workspace support for interaction** |  |  |  |  |  |  |  |  |  |
| Strongly agree /More or less agree | -0.18 | -0.28; -0.09 | <.001 | -0.17 | -0.27; -0.08 | <.001 | -0.13 | -0.23; -0.04 | 0.004 |
| Neither agree nor disagree | -0.07 | -0.18; 0.05 | 0.244 | -0.06 | -0.17; 0.05 | 0.274 | -0.03 | -0.14; 0.08 | 0.578 |
| More or less disagree / Strongly disagree | ref | - |  | ref | - |  | ref | - |  |
| **Ease of workspace switching** |  |  |  |  |  |  |  |  |  |
| Very easy / Quite easy | -0.16 | -0.24; -0.07 | <.001 | -0.15 | -0.23; -0.06 | 0.001 | -0.09 | -0.17; -0.01 | 0.033 |
| Neither easy nor difficult | -0.03 | -0.13; 0.08 | 0.598 | -0.03 | -0.13; 0.08 | 0.611 | 0.00 | -0.10; 0.10 | 0.979 |
| Quite difficult / Very difficult | ref | - |  | ref | - |  | ref | - |  |
| **Access to quiet workspaces** |  |  |  |  |  |  |  |  |  |
| Yes, sufficiently | -0.12 | -0.31; 0.06 | 0.191 | -0.12 | -0.30; 0.07 | 0.215 | -0.08 | -0.26; 0.09 | 0.347 |
| Varyingly, not well enough | -0.02 | -0.20; 0.16 | 0.837 | -0.02 | -0.20; 0.16 | 0.856 | -0.04 | -0.21; 0.14 | 0.665 |
| No, not at all | ref | - |  | ref | - |  | ref | - |  |

Model 1 analyses were adjusted for age, gender, and supervisory position, and telework frequency was added to Model 2 analyses. Model 3 analyses were adjusted for Model 1 covariates (age, gender and supervisory position) and Effort-reward imbalance index.

*N (obs. used) = 779–796, **N= 948–960

| **Supplementary Table 2.** Results from regression models for daily recovery and self-reported work ability with unstandardized adjusted estimates and 95% confidence limits (CL) and p-values. | | | | | | | | | |
| --- | --- | --- | --- | --- | --- | --- | --- | --- | --- |
| **Outcome: Daily recovery** | **Model 1 (N=785–961)** | | | **Model 2 (N=785–961)** | | | **Model 3 (N= 778–954)** | | |
|  | **Estimate** | **95% CL** | **p** | **Estimate** | **95% CL** | **p** | **Estimate** | **95% CL** | **p** |
| **Task privacy**** | 0.35 | 0.26; 0.44 | <.001 | 0.39 | 0.30; 0.48 | <.001 | 0.20 | 0.12; 0.29 | <.001 |
| **Satisfaction with work environment**** | 0.31 | 0.23; 0.39 | <.001 | 0.33 | 0.25; 0.42 | <.001 | 0.20 | 0.12; 0.27 | <.001 |
| **Person-environment fit*** |  |  |  |  |  |  |  |  |  |
| Strongly agree /More or less agree | 1.10 | 0.75; 1.45 | <.001 | 1.13 | 0.78; 1.49 | <.001 | 0.80 | 0.48; 1.13 | <.001 |
| Neither agree nor disagree | 0.91 | 0.41; 1.40 | <.001 | 0.89 | 0.40; 1.39 | <.001 | 0.70 | 0.24; 1.16 | 0.003 |
| More or less disagree / Strongly disagree | ref | - |  | ref | - |  | ref | - |  |
| **Workspace support for interaction**** |  |  |  |  |  |  |  |  |  |
| Strongly agree /More or less agree | 0.88 | 0.54; 1.23 | <.001 | 0.93 | 0.58; 1.27 | <.001 | 0.49 | 0.16; 0.81 | 0.003 |
| Neither agree nor disagree | 0.61 | 0.21; 1.01 | 0.003 | 0.62 | 0.22; 1.02 | 0.003 | 0.26 | -0.11; 0.63 | 0.168 |
| More or less disagree / Strongly disagree | ref | - |  | ref | - |  | ref | - |  |
| **Ease of workspace switching*** |  |  |  |  |  |  |  |  |  |
| Very easy / Quite easy | 0.84 | 0.50; 1.18 | <.001 | 0.86 | 0.52; 1.21 | <.001 | 0.49 | 0.18; 0.81 | 0.002 |
| Neither easy nor difficult | 0.57 | 0.16; 0.99 | 0.007 | 0.57 | 0.16; 0.98 | 0.007 | 0.36 | -0.02; 0.74 | 0.067 |
| Quite difficult / Very difficult | ref | - |  | ref | - |  | ref | - |  |
| **Access to quiet workspaces*** |  |  |  |  |  |  |  |  |  |
| Yes, sufficiently | 1.00 | 0.28; 1.72 | 0.007 | 1.00 | 0.28; 1.72 | 0.007 | 0.78 | 0.11; 1.45 | 0.022 |
| Varyingly, not well enough | 0.58 | -0.13; 1.29 | 0.107 | 0.56 | -0.15; 1.27 | 0.121 | 0.65 | -0.00; 1.31 | 0.051 |
| No, not at all | ref | - |  | ref | - |  | ref | - |  |
| **Outcome: Self-reported work ability** | **Model 1 (N=786–962)** | | | **Model 2 (N=786–962)** | | | **Model 3 (N= 779–955)** | | |
|  | **Estimate** | **95% CL** | **p** | **Estimate** | **95% CL** | **p** | **Estimate** | **95% CL** | **p** |
| **Task privacy^##^** | 0.24 | 0.18; 0.31 | <.001 | 0.25 | 0.19; 0.32 | <.001 | 0.18 | 0.12; 0.25 | <.001 |
| **Satisfaction with work environment^##^** | 0.22 | 0.17; 0.28 | <.001 | 0.23 | 0.17; 0.29 | <.001 | 0.18 | 0.12; 0.24 | <.001 |
| **Person-environment fit^#^** |  |  |  |  |  |  |  |  |  |
| Strongly agree /More or less agree | 0.61 | 0.35; 0.88 | <.001 | 0.61 | 0.35; 0.87 | <.001 | 0.49 | 0.23; 0.76 | <.001 |
| Neither agree nor disagree | 0.55 | 0.19; 0.92 | 0.003 | 0.56 | 0.20; 0.93 | 0.003 | 0.48 | 0.12; 0.85 | 0.009 |
| More or less disagree / Strongly disagree | ref | - |  | ref | - |  | ref | - |  |
| **Workspace support for interaction^##^** |  |  |  |  |  |  |  |  |  |
| Strongly agree /More or less agree | 0.76 | 0.50; 1.02 | <.001 | 0.75 | 0.49; 1.01 | <.001 | 0.60 | 0.35; 0.86 | <.001 |
| Neither agree nor disagree | 0.59 | 0.29; 0.89 | <.001 | 0.58 | 0.28; 0.88 | <.001 | 0.46 | 0.17; 0.76 | 0.002 |
| More or less disagree / Strongly disagree | ref | - |  | ref | - |  | ref | - |  |
| **Ease of workspace switching^#^** |  |  |  |  |  |  |  |  |  |
| Very easy / Quite easy | 0.56 | 0.31; 0.81 | <.0001 | 0.54 | 0.29; 0.80 | <.001 | 0.44 | 0.19; 0.68 | <.001 |
| Neither easy nor difficult | 0.33 | 0.03; 0.64 | 0.033 | 0.33 | 0.02; 0.63 | 0.037 | 0.27 | -0.03; 0.58 | 0.079 |
| Quite difficult / Very difficult | ref | - |  | ref | - |  | ref | - |  |
| **Access to quiet workspaces^#^** |  |  |  |  |  |  |  |  |  |
| Yes, sufficiently | 0.48 | -0.05; 1.01 | 0.075 | 0.46 | -0.07; 1.00 | 0.090 | 0.43 | -0.10; 0.95 | 0.114 |
| Varyingly, not well enough | 0.22 | -0.30; 0.74 | 0.409 | 0.20 | -0.32; 0.73 | 0.447 | 0.28 | -0.24; 0.80 | 0.294 |
| No, not at all | ref | - |  | ref | - |  | ref | - |  |

Model 1 analyses were adjusted for age, gender, and supervisory position, and telework frequency was added to Model 2 analyses. Model 3 analyses were adjusted for Model 1 covariates (age, gender, and supervisory position) and Effort-reward imbalance index.

*N (obs. used) = 778–795, **N= 949–961, ^#^N (obs. used) =779–796, ^##^N=950–962

| **Supplementary Table 3.** Results of regression models for insomnia symptoms and pain index with unstandardized adjusted estimates and 95% confidence limits (CL) and p-values. | | | | | | | | | |
| --- | --- | --- | --- | --- | --- | --- | --- | --- | --- |
| **Outcome: Insomnia symptoms** | **Model 1** (N=789–799) | | | **Model 2** (N=789–799) | | | **Model 3** (N= 782–792) | | |
|  | **Estimate** | **95% CL** | **p** | **Estimate** | **95% CL** | **p** | **Estimate** | **95% CL** | **p** |
| **Task privacy** | -0.12 | -0.16; -0.08 | <.001 | -0.12 | -0.16; -0.08 | <.001 | -0.09 | -0.13; -0.04 | <.001 |
| **Satisfaction with work environment** | -0.10 | -0.14; -0.06 | <.001 | -0.11 | -0.15; -0.07 | <.001 | -0.08 | -0.12; -0.04 | <.001 |
| **Person-environment fit** |  |  |  |  |  |  |  |  |  |
| Strongly agree /More or less agree | -0.32 | -0.47; -0.17 | <.001 | -0.32 | -0.47; -0.17 | <.001 | -0.25 | -0.40; -0.10 | 0.001 |
| Neither agree nor disagree | -0.25 | -0.46; -0.04 | 0.020 | -0.25 | -0.46; -0.04 | 0.020 | -0.20 | -0.40; 0.01 | 0.066 |
| More or less disagree / Strongly disagree | ref | - |  | ref | - |  | ref | - |  |
| **Workspace support for interaction** |  |  |  |  |  |  |  |  |  |
| Strongly agree /More or less agree | -0.16 | -0.32; 0.00 | 0.057 | -0.15 | -0.32; 0.01 | 0.063 | -0.10 | -0.26; 0.06 | 0.203 |
| Neither agree nor disagree | 0.04 | -0.15; 0.23 | 0.680 | 0.04 | -0.15; 0.23 | 0.686 | 0.08 | -0.10; 0.27 | 0.385 |
| More or less disagree / Strongly disagree | ref | - |  | ref | - |  | ref | - |  |
| **Ease of workspace switching** |  |  |  |  |  |  |  |  |  |
| Very easy / Quite easy | -0.11 | -0.26; 0.03 | 0.119 | -0.12 | -0.26; 0.03 | 0.117 | -0.04 | -0.18; 0.10 | 0.602 |
| Neither easy nor difficult | 0.10 | -0.08; 0.27 | 0.281 | 0.09 | -0.08; 0.27 | 0.296 | 0.13 | -0.04; 0.31 | 0.132 |
| Quite difficult / Very difficult | ref | - |  | ref | - |  | ref | - |  |
| **Access to quiet workspaces** |  |  |  |  |  |  |  |  |  |
| Yes, sufficiently | -0.12 | -0.43; 0.18 | 0.425 | -0.13 | -0.44; 0.17 | 0.397 | -0.10 | -0.40; 0.20 | 0.509 |
| Varyingly, not well enough | 0.07 | -0.23; 0.37 | 0.669 | 0.05 | -0.25; 0.36 | 0.724 | 0.02 | -0.27; 0.32 | 0.878 |
| No, not at all | ref | - |  | ref | - |  | ref | - |  |
| **Outcome: Pain Index** | **Model 1** (N=786–796) | | | **Model 2** (N= 786–796) | | | **Model 3** (N= 779–789) | | |
|  | **Estimate** | **95% CL** | **p** | **Estimate** | **95% CL** | **p** | **Estimate** | **95% CL** | **p** |
| **Task privacy** | 0.92 | -0.10; 1.94 | 0.077 | 0.88 | -0.18; 1.95 | 0.104 | 0.60 | -0.46; 1.66 | 0.265 |
| **Satisfaction with work environment** | 0.96 | 0.00; 1.93 | 0.0497 | 0.93 | -0.06; 1.93 | 0.065 | 0.69 | -0.30; 1.68 | 0.174 |
| **Person-environment fit** |  |  |  |  |  |  |  |  |  |
| Strongly agree /More or less agree | 2.24 | -1.48; 5.96 | 0.237 | 2.11 | -1.63; 5.85 | 0.269 | 1.28 | -2.51; 5.06 | 0.508 |
| Neither agree nor disagree | 3.09 | -2.13; 8.31 | 0.245 | 3.15 | -2.08; 8.39 | 0.238 | 2.33 | -2.98; 7.63 | 0.390 |
| More or less disagree / Strongly disagree | ref | - |  | ref | - |  | ref | - |  |
| **Workspace support for interaction** |  |  |  |  |  |  |  |  |  |
| Strongly agree /More or less agree | 1.94 | -2.05; 5.93 | 0.341 | 1.78 | -2.25; 5.82 | 0.386 | 1.19 | -2.84; 5.22 | 0.563 |
| Neither agree nor disagree | -0.63 | -5.27; 4.00 | 0.790 | -0.64 | -5.29; 4.01 | 0.787 | -1.31 | -5.99; 3.36 | 0.582 |
| More or less disagree / Strongly disagree | ref | - |  | ref | - |  | ref | - |  |
| **Ease of workspace switching** |  |  |  |  |  |  |  |  |  |
| Very easy / Quite easy | 3.75 | 0.21; 7.29 | 0.038 | 3.60 | 0.01; 7.19 | 0.049 | 3.13 | -0.48; 6.74 | 0.089 |
| Neither easy nor difficult | 0.67 | -3.68; 5.01 | 0.764 | 0.64 | -3.71; 4.99 | 0.772 | 0.61 | -3.80; 5.01 | 0.787 |
| Quite difficult / Very difficult | ref | - |  | ref | - |  | ref | - |  |
| **Access to quiet workspaces** |  |  |  |  |  |  |  |  |  |
| Yes, sufficiently | 7.37 | -0.12; 14.87 | 0.054 | 7.18 | -0.34; 14.70 | 0.061 | 7.01 | -0.59; 14.61 | 0.071 |
| Varyingly, not well enough | 6.03 | -1.34; 13.39 | 0.109 | 5.97 | -1.42; 13.37 | 0.113 | 6.37 | -1.10; 13.84 | 0.095 |
| No, not at all | ref | - |  | ref | - |  | ref | - |  |

Model 1 analyses were adjusted for age, gender, and supervisory position, and telework frequency was added to Model 2 analyses. Model 3 analyses were adjusted for Model 1 covariates (age, gender, and supervisory position) and Effort-reward imbalance index.
