# Supplementary material for: Perceptions of activity-based offices are associated with employee well-being and self-reported work ability in hybrid work: a cross-sectional study
Source: J Occup Health. 2025 May 20;67(1):uiaf027. doi: 10.1093/joccuh/uiaf027 (PMC12131161; doi:10.1093/joccuh/uiaf027)
Supplement: Web_Material_uiaf027 [file web_material_uiaf027.zip › Supplementary_Data_File_1.docx]

**Supplementary Data, File 1.**

Tulenheimo-Eklund E., Haapakangas A., Hirvonen M., Ruohomäki V., Reijula K. Perceptions of activity-based offices are associated with employee well-being and self-reported work ability in hybrid work: A cross-sectional study. Journal of Occupational Health.

Supplementary Table 1. The table shows the items included in this study.

| Questions | Responses |
| --- | --- |
| Remote work refers to gainful employment that has been agreed with the  employer to be done outside the main work premises. How often do you  normally do remote work?  (adapted from Ruohomäki et al.^1^) | Daily  3–4 days per week  1–2 days per week  2–3 days per month  Less frequently  I don’t do any remote work |
| What kind of workspace do you have at the workplace?  OR (depending on the organization) *:  Do you have a designated workstation at your workplace? | My own office room  Two-person room  Three-person room  Four-person room  Office space for more than four persons, my own workstation (open-plan office)  Activity-based office, shared workstations (no dedicated workstation)  Activity-based office, my own workstation  Other, please specify  I have a designated workstation allocated to me (in a separate office or an open office)  I do not have a designated workstation and I work in shared workspaces (e.g.  activity-based office, flex workstations) |
| What is your opinion on the following statements concerning the work  conditions at the workplace? (adapted from Oldham^2^)   1. I am able to concentrate fully on my   job when I am at work.   1. When I am at the ofﬁce, I can work   with few distractions or interruptions   1. Interruptions at the workplace often prevent me from giving my full   attention to my job | 7-point scale:  1 strongly disagree to 7 strongly agree  1 strongly disagree to 7 strongly agree  1 strongly disagree to 7 strongly agree (reversed for the sum variable) |
| How satisfied are you with your work environment as a whole (at the workplace)?^3^ | 7-point scale:  1 very dissatisfied to 7 very satisfied |
| What is your opinion on the following statements concerning your work  environment?  The work premises are well-suited for carrying out my work tasks.^4^  The work premises support interaction between individuals.^4^ | Strongly disagree  More or less disagree  Neither agree nor disagree  More or less agree  Strongly agree |
| How easy it is for you to find a more suitable workspace during a working  day and go there (e.g. if you need to concentrate or have a confidential  discussion or phone call)? | Very difficult  Quite difficult  Neither easy nor difficult  Quite easy  Very easy |
| At the office, do you have access to quiet workspace for concentrated  work? (adapted from Bodin Danielsson & Theorell^5^) | No, not at all  Varyingly, not well enough  Yes sufficiently |
| The following questions are about how invested you are in your work and  how rewarding the work is. How much do you feel:   1. You focus your abilities and resources   on your work.   1. You are compensated for the work you do in terms income, employee   benefits, etc.   1. You receive recognition or respect for the work you do. 2. You receive personal satisfaction from the work you do^6^ | Very little  Not very much  Neither a little nor a lot  To a considerable extent  To a very great extent |
| How often do you have the feelings or thoughts described by the following  statements?   1. I feel full of energy when I am working 2. I feel enthusiastic about my work 3. I am fully immersed in my work^7^ | Never  A few times a year  Once a month  A few times a month  Once a week  A few times a week  Daily |
| The following statements are related to your work situation and how you  experience this situation. Please state how often each statement applies to  you.   1. At work, I feel mentally exhausted 2. After a day at work, I find it hard to   recover my energy   1. At work, I feel physically exhausted 2. I struggle to find any enthusiasm for   my work   1. I feel a strong aversion towards my   job   1. I’m cynical about what my work   means to others   1. At work, I have trouble staying   focused   1. When I’m working, I have trouble   concentrating   1. I make mistakes in my work because I have my mind on other things 2. At work, I feel unable to control my   emotions   1. I do not recognize myself in the way I react emotionally at work 2. At work I may overreact   unintentionally^8,9^ | Never  Rarely  Sometimes  Often  Always |
| Do you recover from the strain of the working day before the next day?^10,11^ | Scale 0–10:  0 not at all to 10 completely |
| Let’s assume that your work ability at its all-time best would be given 10 points, and 0 points would indicate that you are completely unable to work. What point score would you give your current work ability?  My current work ability compared to the best level of my life^12,13^: | Scale 0–10 |
| The following questions concern your sleep in the past four weeks.   1. Have you had trouble falling asleep? 2. How often have you woken up in the   middle of sleep and it has taken more  than half an hour to fall asleep again  or you have failed to fall asleep at all?   1. How often does your sleep feel   completely unrefreshing?   1. How often has your daytime condition or functional capacity been affected by sleep problems?   (modified and adapted from Hublin et al.^14^) | Less frequently than once in four weeks or never  Less frequently than once a week  1–2 days a week  3–5 days a week  Daily or almost daily |
| How much bodily pain have you had during the past 4 weeks?  During the past 4 weeks, how much did pain interfere with your normal  work (including both work outside the home and housework)?^15^ | None  Very mild  Mild  Moderate  Severe  Very severe  Not at all  A little bit  Moderately  Quite a bit  Extremely |
| Year of birth |  |
| Gender | Female  Male  Other  I do not wish to say |
| What is your level of education? | Elementary school or primary school  Middle school  Vocational school or similar  General upper secondary school / high school  Post-secondary education  Bachelor’s degree (university of applied sciences or similar)  Master’s degree (master or similar)  Post-graduate (licentiate or doctor) |
| Are you in a supervisory position? | No  Yes |

*The latter question was for one organization where all the respondents worked in ABO (information from observations)

1. Ruohomäki V, Vuorento, M., Kaila-Kangas, L., Laitinen, J., Joensuu, M. and Soikkanen, A. . Työn uudet muodot ja työkyvystä huolehtiminen – terveyskäyttäytyminen etätyössä. (New forms of work and ensuring work ability – health-related behaviour in remote work, in Finnish). 2023;Tietoa työstä. Helsinki: Työterveyslaitos.

2. Oldham GR. Effects of changes in workspace partitions and spatial density on employee reactions: A quasi-experiment. *Journal of Applied Psychology*. 1988;73(2):253-258. doi:10.1037/0021-9010.73.2.253

3. Lahtinen M, Ruohomäki V, Haapakangas A, Reijula K. Developmental needs of workplace design practices. *Intelligent Buildings International*. 2015/10/02 2015;7(4):198-214. doi:10.1080/17508975.2014.1001315

4. Ruohomäki V, Haapakangas A, Lahtinen M. Tilat työn mukaisiksi: Työn analyysi ja koettu sisäympäristö yliopistossa (Spaces to match work. An analysis of work and the perceived indoor environment at a university, in Finnish). 2013;Sisäilmastoseminaari. Sisäilmayhdistys ja Aalto-yliopisto, SIY raportti, 31, pp. 135-140.

5. Bodin Danielsson C, Theorell T. Office Employees’ Perception of Workspace Contribution: A Gender and Office Design Perspective. *Environment and Behavior*. 2019;51(9-10):995-1026. doi:10.1177/0013916518759146

6. Kivimäki M, Vahtera J, Elovainio M, Virtanen M, Siegrist J. Effort-reward imbalance, procedural injustice and relational injustice as psychosocial predictors of health: complementary or redundant models? *Occupational and Environmental Medicine*. 2007;64(10):659. doi:10.1136/oem.2006.031310

7. Schaufeli WB, Shimazu A, Hakanen J, Salanova M, De Witte H. An Ultra-Short Measure for Work Engagement. *European Journal of Psychological Assessment*. 2017;35(4):577-591. doi:10.1027/1015-5759/a000430

8. Hadžibajramović E, Schaufeli W, De Witte H. Shortening of the Burnout Assessment Tool (BAT)-from 23 to 12 items using content and Rasch analysis. *BMC Public Health*. Mar 22 2022;22(1):560. doi:10.1186/s12889-022-12946-y

9. Schaufeli WB, De Witte H, Hakanen JJ, Kaltiainen J, Kok R. How to assess severe burnout? Cutoff points for the Burnout Assessment Tool (BAT) based on three European samples. *Scand J Work Environ Health*. May 1 2023;49(4):293-302. doi:10.5271/sjweh.4093

10. Selander K, Korkiakangas E, Toivanen M, et al. Engaging Leadership and Psychological Safety as Moderators of the Relationship between Strain and Work Recovery: A Cross-Sectional Study of HSS Employees. *Healthcare (Basel)*. Apr 5 2023;11(7). doi:10.3390/healthcare11071045

11. Kinnunen U, Feldt T, Siltaloppi M, Sonnentag S. Job demands-resources model in the context of recovery: Testing recovery experiences as mediators. *European Journal of Work and Organizational Psychology*. 2011;20(6):805-832. doi:10.1080/1359432x.2010.524411

12. Tuomi K, Ilmarinen J, Martikainen R, Aalto L, Klockars M. Aging, work, life-style and work ability among Finnish municipal workers in 1981-1992. *Scand J Work Environ Health*. 1997;23 Suppl 1:58-65.

13. Ahlstrom L, Grimby-Ekman A, Hagberg M, Dellve L. The work ability index and single-item question: associations with sick leave, symptoms, and health – a prospective study of women on long-term sick leave. *Scandinavian Journal of Work, Environment & Health*. September 36 2010;(5):404-412. doi:10.5271/sjweh.2917

14. Hublin C, Lehtovirta M, Partinen M, Koskenvuo M, Kaprio J. Changes in sleep quality with age–a 36-year follow-up study of Finnish working-aged adults. *Journal of Sleep Research*. 2018;27(4):e12623. doi:10.1111/jsr.12623

15. Hays RD, Sherbourne CD, Mazel RM. The RAND 36-Item Health Survey 1.0. *Health Econ*. Oct 1993;2(3):217-27. doi:10.1002/hec.4730020305
